# Discoveries Interview: Prof. Jack Lawler on the discovery and significance of thrombospondins

**DOI:** 10.15190/d.2014.4

**Published:** 2014-03-18

**Keywords:** Jack Lawler, thrombospondins, Harvard, BIDMC, Division of Cancer Biology and Angiogenesis

**Figure 1:**
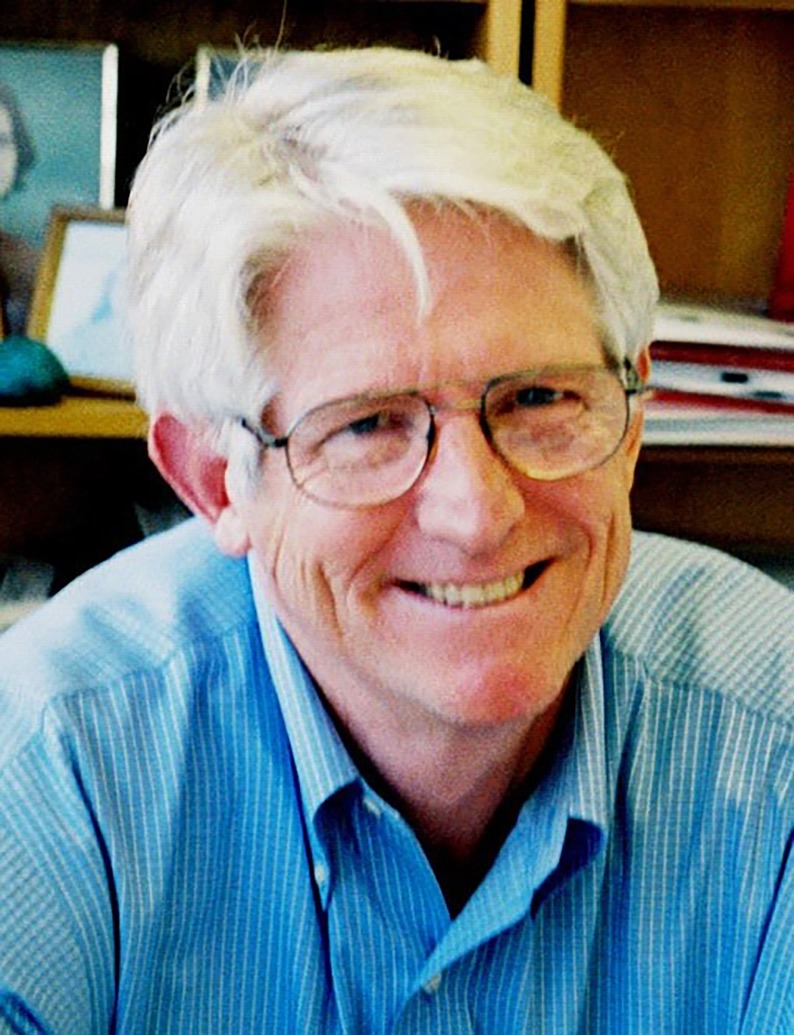
Professor Jack Lawler

**Jack Lawler Ph.D. **is Professor of Pathology at Harvard Medical School and Director of Experimental Pathology at the Beth Israel Deaconess Medical Center, Boston, Mass. Additionally, Dr. Lawler is Co-Director of the Training Grant in Angiogenesis and Inflammation in the Department of Pathology at the Beth Israel Deaconess Medical Center and is the Leader of the Angiogenesis, Invasion and Metastasis Discipline-based Working Group of the Dana Farber/Harvard Cancer Center.

Dr. Lawler received his B.S. in Physics from Villanova University in 1971 and his Ph.D. in Physics from Boston College in 1976. He was a post-doctoral fellow at the Dana Farber Cancer Institute in the Structural Biology Laboratory where he performed the first purification and biochemical characterization of thrombospondin-1. In 1982, Dr. Lawler became Assistant Professor in the Department of Medicine at Tufts University Medical School and St. Elizabeth’s Hospital, Boston. While Dr. Lawler was a Visiting Scientist in Richard Hynes’ lab at MIT, he cloned and sequenced thrombospondin-1 and engineered the thrombospondin-1-null mouse. In 1988, Dr. Lawler became Associate Professor of Pathology at Harvard Medical School and Brigham and Women’s Hospital. He has published over 200 articles and co-authored the book *“The Thrombospondin Gene Family”. *Dr. Lawler has also served on the editorial boards of the *Journal of Cellular and Molecular Medicine, Current Drug Targets*, *Journal of Cell Communication and Signaling, and Discoveries.* The NIH has continuously supported his work for three decades.

## 1. Can you define in simple terms the thrombospondin gene family and explain how the members regulate cellular proliferation, migration, differentiation, and apoptosis?

The members of the thrombospondin (TSP) gene family function in the endoplasmic reticulum and the plasma membrane to assemble protein complexes that guide cellular behavior and response to stimuli. In addition, the TSPs probably facilitate the assembly of the extracellular matrix. Through a wide range of interactions with other proteins and proteoglycans, TSPs participate in synapse formation, angiogenesis, inflammation, wound healing, and development. The precise molecular mechanisms through which TSPs participate in these processes are active areas of investigation. A recent analysis of the TSP interactome revealed 83 binding partners^1^ .The TSPs probably engage multiple partners simultaneously because the domains for these interactions are distributed throughout the TSPs and the proteins are multimeric. TSP-1 and -2 are trimeric, while TSP-3, -4, and -5 (also called cartilage oligomeric matrix protein) are pentameric. TSP-1 is the best characterized because it was the first to be identified and is readily purified from blood platelets^2^.

## 2. How were the thrombospondins discovered and how has our knowledge about them evolved over time?

As a graduate student, I observed that platelets contain a 450,000-dalton protein that is secreted in response to thrombin, hence the name thrombospondin^3^. The rest of my career has been devoted to the characterization of the structural and functional properties of the members of the TSP gene family. It is fair to say that such a journey of pure discovery would be impossible to embark upon today because grant reviewers would be left pondering the *impact* of such an endeavor.

The scope and breadth of the field has expanded continuously since those early days. In the mid 80’s it was established that TSP-1 was made by a wide variety of cells and tissue. The complexity of TSP-1’s effects on cells and disease processes increased considerably with the realization that TSP-1 activates the pleotropic protein latent transforming growth factor β (TGFβ)^4^. The advent of cloning and sequencing techniques led to the realization that there are five members of the gene family, each with its distinct pattern of tissue expression. The sequence of TSP-1 led to the identification of the thrombospondin type 1 repeat, a novel protein fold that is present in a variety of proteins that are present in many species^5^. The knowledge of the sequence also led to the realization that a p53-regulated anti-angiogenic protein produced by fibroblasts from Li-Fraumeni patients was a proteolytic fragment of TSP-1^6^. The development of techniques for gene deletion through homologous recombination enabled the development of mice that lacked single and multiple TSPs. The fact that all of these mice are viable has facilitated the analysis of the role of TSPs in wound healing, vascular and heart disease, blood pressure, coagulation and thrombosis, inflammation, synaptogenesis, angiogenesis, skeletal development and inherited diseases, nitric oxide regulation, and cancer.

## 3. What will the field look like in 5-10 years?

The function of TSPs in a wide range of biological processes will b appreciated in the future. These studies will doubtless include new fields that we don’t appreciate as being important today. The continuous discovery of new roles for TSPs over the past thirty years has been perhaps the most exciting aspect of the study. New functions for the TSPs in the central and peripheral nervous systems will likely be revealed soon.

The TSPs are remarkable in their ability to bind calcium ions with each subunit containing about 30 calcium-binding sites. The importance of these calcium ions for TSP function is largely unknown. Calcium-dependent folding of TSP-1 appears to be required for secretion. Since TSPs can initiate an unfolded protein response, it is possible that the cell uses the folding of the calcium-dependent structures as a way to monitor the fidelity of protein folding in general. It is also possible that TSPs buffer calcium in the ER or enrich it at the plasma membrane. In the not too distant future, we will have a better understanding of the role of calcium in TSP function.

TSP-1 was the first endogenous protein inhibitor of angiogenesis to be identified. Whereas considerable progress has been made in determining the molecular mechanism underlying this activity, the use of TSP-1 in the clinic is still in its infancy. It should be possible to formulate the type 1 repeats of TSP-1 and -2 so that they can be used to inhibit pathological angiogenesis. Conversely, it should be possible to increase angiogenesis during wound healing and islet transplantation by suppressing TSP-1 and -2 expression. It is reasonable to expect that a therapeutic that is based on the type 1 repeats of TSP-1 and -2 will be in the clinic in the near future for the treatment of cancer. It was certainly logical to start with antagonists of the vascular endothelial growth factor (VEGF) pathway when seeking to shift the angiogenic balance toward inhibition. This approach was validated by the remarkable success of Lucentis for the treatment of wet age-related macular degeneration. However, the results of recent clinical trials for anti-VEGF treatment of cancer have led some to question the efficacy and cost-effectiveness of the approach. These modest results of anti-VEGF therapy for cancer are not surprising given the complexity of tumor angiogenesis and the instability of the cancer cell genome. VEGF is only one of the pro-angiogenic factors in the tumor microenvironment. It should be effective to treat cancer patients with a therapeutic based on the type 1 repeats, which have been shown to inhibit endothelial cell response to IL-8, bFGF, PDGF and aFGF, as well as VEGF. Thus, it is logical to predict that a cocktail of angiogenic inhibitors will be needed to effectively inhibit tumor angiogenesis in much the same way that drug cocktails are used for chemotherapeutics and anti-viral drugs. TSPs or their active domains may be delivered using cell-based strategies or through incorporation into Fc fusion proteins.

## 4. What advice do you have for young scientists?

Don’t try to go it alone. Scientific research is most enjoyable when it is done in collaboration with colleagues who have complementary expertise. These collaborations can save considerable time in the generation of reagents and models. National and international meetings are an excellent way to make connections and to develop an identity in a particular field of research. It is important to be active in the societies that sponsor these meetings and in the community of scientists in your field. The connections that are made in your field become very important during peer-review. The need for support includes your institution. It is very important to work in a department and at an institution that appreciates your work and is willing to support it.
